# G Protein-Coupled Receptors in Cancer

**DOI:** 10.3390/ijms17081320

**Published:** 2016-08-12

**Authors:** Rachel Bar-Shavit, Myriam Maoz, Arun Kancharla, Jeetendra Kumar Nag, Daniel Agranovich, Sorina Grisaru-Granovsky, Beatrice Uziely

**Affiliations:** 1 Sharett Institute of Oncology, Hadassah-Hebrew University Medical Center, Jerusalem 91120, Israel; Myriamm@hadassah.org.il (M.M.); kancharlarn@gmail.com (A.K.); jeetendr.nag@mail.huji.ac.il (J.K.N.); Danyaag@gmail.com (D.A.); Beatrice@hadassah.org.il (B.U.); 2 Department of Obstetrics and Gynecology, Shaare-Zedek Medical Center, Jerusalem 91031, Israel; sorina@szmc.org.il

**Keywords:** G protein-coupled receptors (GPCRs), protease, protease-activated receptor, protease-activated receptors (PARs), PH-domain, oncogenes, cancer, LPA(1-6), CXCR4, Wnt/β-catenin, Hippo/YAP

## Abstract

Despite the fact that G protein-coupled receptors (GPCRs) are the largest signal-conveying receptor family and mediate many physiological processes, their role in tumor biology is underappreciated. Numerous lines of evidence now associate GPCRs and their downstream signaling targets in cancer growth and development. Indeed, GPCRs control many features of tumorigenesis, including immune cell-mediated functions, proliferation, invasion and survival at the secondary site. Technological advances have further substantiated GPCR modifications in human tumors. Among these are point mutations, gene overexpression, GPCR silencing by promoter methylation and the number of gene copies. At this point, it is imperative to elucidate specific signaling pathways of “cancer driver” GPCRs. Emerging data on GPCR biology point to functional selectivity and “biased agonism”; hence, there is a diminishing enthusiasm for the concept of “one drug per GPCR target” and increasing interest in the identification of several drug options. Therefore, determining the appropriate context-dependent conformation of a functional GPCR as well as the contribution of GPCR alterations to cancer development remain significant challenges for the discovery of dominant cancer genes and the development of targeted therapeutics.

## 1. Introduction

G protein-coupled receptors (GPCRs) comprise the largest family of cell surface receptors in the human genome, regulating a plethora of physiological responses and serving as frequent drug targets. Despite their broad physiological functions and associated disease processes, which have resulted in their designation as favorable sites for pharmacological drug development, their role in tumor biology is underappreciated. Conformational changes take place after ligand binding, inducing the activation of complex signaling schemes that in turn lead to a cell response. Canonical GPCR agonist activation involves the recruitment of G proteins followed by the phosphorylation of the receptor by G protein-coupled receptor kinase (GRK), “allowing” the binding of β-arrestin 1 and 2 (e.g., referring to arrestin 2, the first non-visual arrestin, and 3 (the second cloned non-visual arrestin), respectively) and subsequent internalization into endosomes. Internalized receptors can either recycle back to the cell surface or undergo degradation. In the past, the “two-state” receptor model (inactive and active states) was widely accepted to explain GPCR function; however, a more intricate and complex “multi-state” model entailing high GPCR conformational dynamics is now favored. GPCRs are pleiotropic with respect to the cell signal proteins they activate within a cell, and therefore more than one conformation of a receptor exists. Hence, different ligands can induce distinct receptor conformational states following activation, initiating several specific downstream signaling profiles. Several conformational changes in a single GPCR, eliciting discrete signaling pathways, is termed “biased agonism” [1,2,3,4]. As an example, it has been demonstrated that, in addition to regulating the GPCR signaling that induces internalization and desensitization, β-arrestin 1 and 2 are also capable of initiating distinct signals on their own [5]. For example, in the activation of the endothelin receptor (ETA) receptor via endothelin (ET-1/ET_A_R) in epithelial ovarian cancer, β-arrestin 1 is required to maintain NFκB transcriptional activity in response to endothelin receptor A (ET_A_R) activation. In addition, in response to ET_A_R activation (via ET-1), β-arrestin 1 increases its nuclear localization and binds to nuclear β-catenin, thereby enhancing β-catenin transcriptional activity, a central path in ovarian cancer [6,7].

Four main groups of GPCRs are recognized and classified according to their pharmacological properties by the guidelines of the International Union of Basic and Clinical Pharmacology: Class A is rhodopsin-like; Class B is secretin-like; Class C is comprised of metabotropic glutamate/pheromone; and Class D is comprised of frizzled receptors. Class A is the largest and best-studied family, and includes several members that play a major part in tumor biology, for example protease-activated receptors, or protease-activated receptors (PARs). Class A has been further subdivided into four groups: α, β, γ, and δ. The δ group contains, among others, the leucine-rich repeat-containing receptors (LGRs) including LGR5, a *bona fide* stem cell marker for colon and breast tissues.

While GPCRs regulate many aspects of tumorigenesis as well as many cancer-associated signaling pathways [8,9], only a few drugs aiming to inhibit GPCRs are currently used in cancer. Genome-wide major analyses of multiple human tumors have exposed novel GPCRs that are modified in cancer and might be potential candidates for cancer drug development. Importantly, it is imperative to differentiate between cancer driver genes and bystanders to identify valid targets for personalized medicine in the future. Indeed, pharmacological treatments targeting GPCRs will become increasingly attractive as more data associating GPCRs with cancer emerges. Understanding the molecular machinery of GPCRs in tumor development may contribute to tumor-related GPCR drug development. In this review we discuss recent advances in cancer-associated GPCRs and signal proteins such PARs, chemokine receptors, Gα12/13 proteins, lysophosphatidic acid (LPA), and GPCR-mediated pathways such as the WNT and Hippo signaling pathways. We also describe potential drug design targets such as the pleckstrin-homology (PH) binding motifs that were found and characterized in PAR-implicated tumor biology.

## 2. Biasing towards Specific G-Proteins in Cancer

The structural signature of seven transmembrane domains that couple to G proteins for signaling are among the common themes in GPCRs. G proteins are divided into four main sub-groups: Gαs, Gαq/11, Gαi/o and Gα12/13 which are associated selectively, upon ligand activation, to initiate a potential downstream signaling pathway. G proteins are composed of three subunits, Gα, Gβ and Gγ which are located in the inner part of the plasma membrane. Upon ligand binding the signal is transmitted through conformational changes, which consequently result in the initiation of the G protein cycle of association. In fact, GPCRs function as guanine nucleotide exchange factors for α subunit of the G protein, promoting the exchange of bound GDP for GTP-α. Bound GTP-α allows the switch from an inactive state (of the bound trimeric G proteins) to an active status of the GTP-α subunit and the release of βγ subunits. These βγ subunits consequently activate downstream signaling partners such as Src, phospholipase C, adenylyl cyclase, phosphodiesterases and ion channels. The cycle is terminated by the hydrolysis of α subunit-bound GTP to GDP, and its re-association with βγ G proteins for turning off the signal.

A significant feature of a biased GPCR ligand is the ability to activate either of the G protein subfamilies, Gαs, Gαq/11, Gαi/o or Gα12/13, for selectively harnessing and recruiting a specifically selected downstream signal pathway. While most of the G proteins are not associated with cancer, the Gα12/13 family is connected with cell transformation (e.g., fibroblasts) [10,11], thus directing toward tumor-related processes. Gα12/13 family members may be also involved in the control of the Rho-dependent formation of stress fibers, the Jun kinase/stress-activated protein kinase pathway, and the Na^+^/H^+^ exchanger [12,13,14].

Genetic ablation of Gα13 in mice results in embryonic lethality at a stage when gastrulation is already completed (about embryonic day 9.5). On the other hand, the ablation of Gα12, the other family member, results in viable mice exhibiting a normal phenotype. This genetic outcome points to distinct roles of the Gα12/13 family members. In addition, a defective assembly of the vascular system, which is prominent mostly in the yolk sac and in the head mesenchyme, was also demonstrated in Gα13-deficient mouse embryos [15].

LPA receptors are coupled to Gαq/11, Gαi and Gα12/13 [16,17,18]. In NIH 3T3 and neuroblastoma B103 cells, the LPA3 receptor is coupled to Gαi, leading to Ras-GTP accumulation of mitogen-activated protein kinase (MAPK) activation and enhanced cell proliferation [19,20]. LPA1, LPA2 and LPA3 receptors in PC12 cells are coupled to Gαq/11 following neurokinin A or endothelin binding to these receptors, thereby inducing signaling via tyrosine kinase c-Src but not Ras and β-catenin via β-arrestin 1 [7,21]. Other examples in PC12 cells demonstrate that LPA1-3 binds to G*α*12/13 [22]

G proteins may direct biased agonism also in various metabolic disease systems (other than cancer) as for example, coupling of GPR109A to Gαi/o, following biased ligand activation, leading to induced levels of high-density lipoprotein and consequently to a decrease in triacylglycerol levels. As a result it leads to a significant decrease in cardiovascular morbidity and mortality [23]. Protease-activated receptor 1 (PAR_1_) as a member of the GPCR family, binds also to various heterotrimeric G protein subtypes within a cell, promoting different cellular functions. For example, as recently elegantly demonstrated, N-linked glycosylation of PAR_1_ at extracellular loop 2 (ECL2) favors coupling to Gα12/13-dependent Rho activation. In contrast, a mutant form lacking in glycosylation at the ECL2 region couples more efficiently to Gαq, mediating phosphoinositide (PI) signaling [24]. Traditionally, PAR_1_ stimulates phospholipase C (PLC)-induced PI signaling via Gαq, while inhibiting Gαi-mediated adenylate cyclase signaling. Biased agonism could activate not only different G protein subtypes, but also stimulate an alternate system to G proteins as signaling transducers, such as β-arrestins [25]. The β-arrestin bias may, in some cases, confer positive effects and rather than mediating internalization and degradation, ir directs the recruitment, activation, and scaffolding of cytoplasmic signaling complexes via β-arrestins 1 and 2. For example, a β-arrestin–biased ligand, PTH (parathyroid hormone), promotes bone formation and homeostasis, thus reducing hypercalcemia of malignancy and osteoporosis [26]. Nevertheless, the molecular mechanism of biased ligands between β-arrestin and the G protein pathways are not yet known. β-arrestin has also been shown as critical for Wnt/β-catenin signal transduction. The axis of Dishevelled (DVL)-β-arrestin interaction is important for the Wnt/β-catenin signaling. It has been demonstrated that in mouse embryonic fibroblasts (MEFs), genetic ablation of β-arrestin 1 and/or 2 impairs wnt-3A-induced activation of DVL and β-catenin signaling [27]. In ovarian cancer the activation of the endothelin-A receptor (ET_A_R) by endothelin-1 (ET-1) plays a central role in ovarian cancer progression. Silencing of both β-arrestin 1 and β-arrestin 2 inhibits these receptors’ (e.g., ET_A_R) signaling, reducing, among others, Src and serine/threonine kinase Akt (AKT) activation finally affecting the β-catenin pathway [28].

## 3. G Protein-Coupled Receptor (GPCR) and Oncogenicity

The first link between cellular transformation and GPCRs was discovered in 1986 with the identification of the MAS oncogene [29]. The *mas* gene product exhibits properties characteristic of GPCRs, including seven pass of the membrane, and is made up of 325 amino acids. MAS is the receptor for the metabolite angiotensin-(1–7) (Ang-(1–7)), which is formed by angiotensin-converting enzyme 2 on angiotensin II and functions as a vasodilator and antiproliferative agent [30]. Using a cDNA expression library screen for oncogenes revealed two other GPCRs with oncogenic properties, namely G2A and PAR_1_. The role of the PAR family (whereby PAR_1_ is the first and prototype member) in cancer biology will be discussed below.

Another oncogene, G2A, was independently identified by Kay and colleagues [31] and Witte et al., [32]. They showed [31] that forced expression of G2A in NIH3T3 cells led to an increased number of cells in the G2/M cell-cycle phase; therefore, they identified this gene as G2A (G2 accumulation). In addition, overexpression of G2A antagonized the activity of Bcr-Abl in Rat-1 fibroblasts and in mouse bone marrow cells, inducing increased cell proliferation. Therefore, G2A may either promote or antagonize growth, depending on the cellular context. G2A mRNA is expressed most often in hematopoietic tissue and cell lines. No mutations were found in G2A and no known ligand for G2A has been identified. It is not yet known whether G2A transformation is ligand-independent or takes place as a consequence of an unknown serum ligand or a NIH3T3 cell-residing ligand.

Whitehead and co-workers identified PAR_1_, which encodes the prototype member of the PAR family. Using the anchorage-independent foci formation assay in NIH3T3 cells, they demonstrated in two independent screens [33,34] that overexpression of naïve human PAR_1_ caused a similar transforming activity; both loss of anchorage- and serum-dependent growth were observed in NIH 3T3 cells with PAR_1_ overexpression. This activity was described in addition to its potent foci-forming activities. We have demonstrated that PAR_1_ overexpression induces invasion in pathological cancer cells as well as in the physiological invasion process of placenta implantation into the uterus deciduas [35]. Significantly, no mutations have been found in any of the PAR family members. Its transforming activity is attributed to receptor overexpression in malignant epithelial cells as compared to no expression in normal epithelium. It is postulated that PAR-transforming activities are ligand-dependent, and that they are seen in correlation with the plethora of serine-protease present in the dynamic milieu of a tumor microenvironment.

## 4. *Gep* Oncogenes

Members of the Gα12 family, Gα12 and Gα13, were isolated at first as the *gep* oncogenes with transforming capabilities [10,36]. These G proteins regulate various cellular processes, among which are migration, proliferation, transformation, platelet aggregation, neurite retraction, and actin-stress fiber formation [37,38,39]. Gα12 and Gα13 belong to the large family of G proteins consisting of α, β, and γ subunits. They induce signals from GPCRs to intracellular effectors [40,41,42]. The α-subunit is a protein of 37–42 kDa including the guanine nucleotide-binding site and the intrinsic GTPase activity. Ligand-activated GPCRs catalyze the exchange of bound GDP to GTP in the α subunit. GTP-bound subunits stimulate distinct downstream effectors. A constitutively activated mutant of Gα_12_ (Gα_12_QL), as well as Gα_13_QL, effectively transform NIH3T3 fibroblasts, as determined via foci-forming activities [43,44,45]. They also control small GTP-binding proteins (i.e., the Ras and Rho family) and have an impact on the activity of several transcription factors such as serum response factor (SRF), activating protein 1 (AP-1), the nuclear factor of activated T cells (NFAT), a signal transducer and activator of transcription 3 (STAT3), and nuclear factor-kB [46,47,48,49]. The small GTPases Rho and Rac play critical roles in communicating Gα12/Gα13 signaling through the Rho family of GTPases [47,48]. Indeed, GPCR ligands such as thrombin, LPA, and S1P are involved in stimulating tumor growth and invasion via coupling of their cognate receptors to Gα12/13 proteins. Hence, a considerable challenge is the identification of anticancer drugs targeting Gα12/13, PARs, LPA and SIP receptors.

## 5. Lysophosphatidic Acid (LPA) Receptors in Tumor Biology

The phospholipid LPA signals via no fewer than six receptors (LPA1–LPA6) belonging to the GPCR family. LPA1–LPA3 (also known as EDG2, EDG4, and EDG7, respectively) are expressed by endothelial differentiation genes [17]. LPA4 (also known as GPR23/P2y9), LPA5 (GPR92), and LPA6 (GPR87) are included within the purinergic family of GPCRs [50,51,52]. Inducing LPA and/or an aberrant expression of its receptors may lead to cancer initiation and progression [53,54]. This has been demonstrated in breast cancer [55] and ovarian cancer [56], where LPA acts via activation of the Rho-dependent transduction pathway to elicit migration and tumor formation. In addition to the manipulation of LPA activity via cognate receptors, novel and selective agonists and antagonists might be employed therapeutically through understanding the differences between LPA1-3 receptors, since LPA biosynthesis is considered a feasible target for therapeutics [57].

## 6. Chemokine Receptors

Cancer cells that metastasize preferentially to specific organs via the blood and lymphatic vessels present a great challenge in cancer eradication. One family of GPCRs that is closely linked to tumor metastasis is the chemokine receptors. Chemokines enhance the motility and survival of cancer cells in the vicinity and milieu of a tumor following their local release in either an autocrine or paracrine fashion into the microenvironment of tumor-surrounding regions [58]. Among these are chemokines that are involved in metastatic cancer cell homing [59] as well as cancer cell growth and survival [60], such as chemokine receptors CCR7 and CCR10. Local chemokine generation in the tumor milieu may recruit macrophages and leukocytes, which can then induce the release of matrix metalloproteases (MMPs) promoting tumor cell survival, growth, and invasion as well as improving the cytokine-rich microenvironment. CXCR4 is a well-documented chemokine receptor driving cancer metastasis. Moreover, cells in the most frequent sites of metastasis, including the lungs, bone marrow, lymph nodes, and liver, express the chemokine ligand CXCL12/SDF-1 [61]. Tumor cells frequently express high levels of CXCR4, facilitating cell growth, survival, and migratory capability. For example, while CXCR4 is not found in normal breast tissues, it is rather overexpressed in breast cancer cells [62] and a marked inhibition of breast cancer metastatic spread is achieved by inhibiting CXCR4 [58,62,63]. However, treatment with CXCR4 inhibitors requires caution, since CXCR4 inhibition induces progenitor/stem cell mobilization from the bone marrow. Hypoxia-inducible factor-1 (HIF-1α), which is activated by hypoxia, increases CXCR4 transcription [64]. In highly aggressive basal-like breast cancer cells, CXCR4 may also couple to Gα12/13 when Gα13 protein is highly upregulated, and consequently drives spread via lymphatic vessels and site-specific metastasis in a Gα12/13-RhoA-dependent manner [65]. This molecular machinery is mediated similarly via PARs and LPA, all of which may serve as possible targets for metastasis prevention and treatment.

## 7. Wnt Signaling

**Scheme 1:**
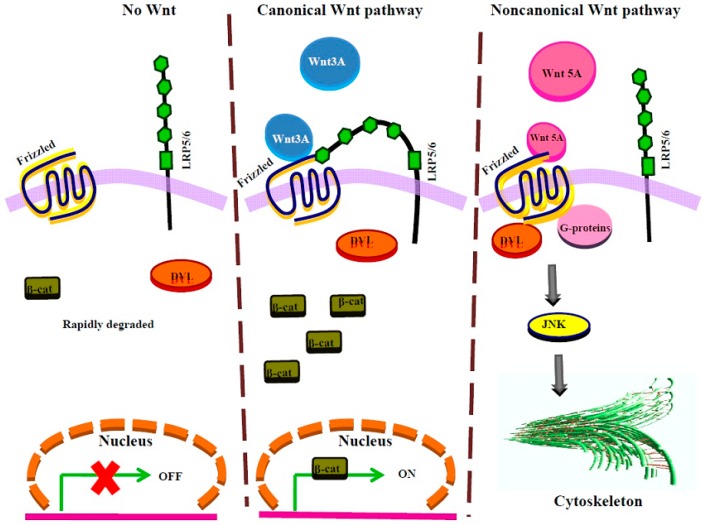
Illustration of Wnt/β-catenin canonical and noncanonical pathways. In the presence of a Wnt ligand (e.g., Wnt 3A), Frizzled receptor (Fz) co-associates with LRP5/6, leading to stabilization of β-catenin. In contrast, in the absence of a Wnt ligand, β-catenin is rapidly degraded via the proteasomal compartment. Stabilized β-catenin enters the nuclei and functions as a co-transcription factor, inducing a spectrum of gene signature downstream. Noncanonical Wnt signaling (e.g., Wnt 5a) is mediated via Fz affecting, among others, activation of JNK and the cytoskeleton. Rred cross: Inhibition of signal cascade.

Wnt proteins were first identified in Drosophila, from which their name was coined. They are critically involved in controlling both normal development and tissue homeostasis, as well as pathological processes such as cancer. Intensive efforts have been made to unravel the Wnt (Wingless ad INT-1) signaling pathway. Frizzled (Fz) receptors are a subgroup of GPCRs that play a pivotal role in development, tissue homeostasis, and cancer, serving as receptors for Wnts. Wnt signaling stabilizes β-catenin through Fz and the low-density lipoprotein-related protein 6 (LRP6) receptor complex that antagonizes the β-catenin “destruction complex”. The canonical Wnt pathway refers to the activation of the highly conserved Wnt/β-catenin signaling pathway, involving the stabilization of β-catenin via Wnt binding to Fz cell surface receptors and LRP5/6 co-receptors. In the absence of Wnt, the key effector of this pathway, β-catenin, is continuously degraded by the “degradation complex”. This complex is comprised of Axin, adenomatis polyposis coli (APC), glycogen synthase kinase3β (GSK3β), casein kinase1α (CK1α), and the E3 ubiquitin ligase subunit β-TrCP1. Axin provides a scaffolding site for GSK3β to phosphorylate the N-terminal portion of β-catenin (after priming by CK1α), thereby generating a phosphorylated form of β-catenin that is recognized by the ubiquitin ligase adaptor β-TrCP [66,67]. Wnt stimulation dismantles the degradation complex, leading to the accumulation of unphosphorylated β-catenin. Once β-catenin is stabilized, it is translocated to the cell nucleus. There it alters the activity of the lymphoid enhancer factor (Lef)/T cell factor (Tcf) family members. The Lef/Tcf family belongs to HMG-box transcription factors and acts as a transcriptional switch, recruiting various chromatin modifiers and remodelers to Lef/Tcf target genes, inducing expression of an array of genes downstream (Scheme 1; [67,68]). A wide range of cancers exhibit hyperactive stabilized β-catenin, either because of oncogenic mutations in its N-terminal phosphorylation site or through mutational inactivation of APC or Axin, its negative regulators [68,69]. Activated β-catenin can be oncogenic, driving the onset of a wide spectrum of carcinomas. Noncanonical Wnt signaling does not involve β-catenin/Tcf activity and does not utilize the LRP5/6 co-receptor. For example, Wnt5a/b are prototypes of this Wnt pathway [70]. In vertebrates, noncanonical Wnt signaling is involved in planar cell polarity (PCP), dorsoventral patterning, tissue regeneration, convergent extension movements, and tumorigenesis. Throughout these processes, alternative Wnt signaling induces the small G protein Rho. Rho activates Rho-associated kinase (ROCK), which is one of the major regulators of the cytoskeleton and, in-general, this noncanonical signaling antagonizes canonical Wnt/β-catenin signaling. Fz receptors transduce both Wnt/β-catenin and noncanonical Wnt signaling. An interesting yet unresolved aspect of Fz is the involvement of G proteins. While Gα proteins have been shown to alter Wnt signaling in some studies [71,72,73], other research has failed to identify Gα proteins as essential components of Wnt/β-catenin signaling [74,75]. Thus, the involvement of G proteins in Wnt signaling pathways is yet an open, controversial issue.

## 8. GPCR Regulation of Hippo Signaling Pathway

**Scheme 2:**
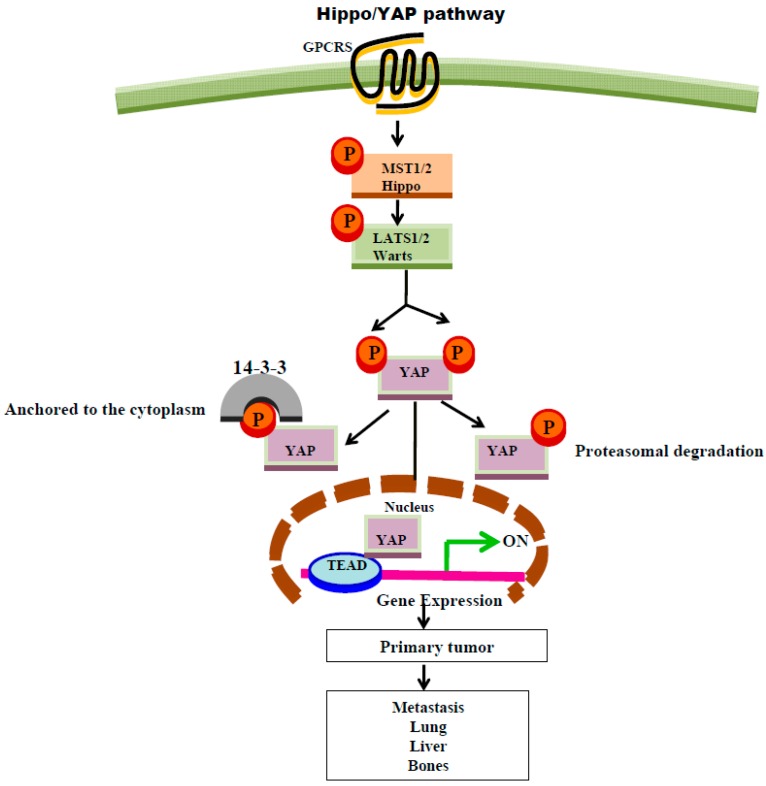
The Hippo/YAP pathway is physiologically initiated via GPCRs. The Hippo pathway takes place following the phosphorylation of Ltats1/2 by Mst1/2 which leads to the phosphorylation of YAP and its anchoring localization in the cytoplasmic compartment. YAP is activated by inhibition of the Hippo pathway via the de-phosphorylation of YAP, resulting in YAP nuclear localization and its function as a co-transcription factor.

The Hippo-YAP/TAZ pathway has emerged as a major conserved path that integrates diverse stimuli in a broad range of functions, including control of cell growth and organ size as well as mechanical and cytoskeletal proteins, apico-basolateral polarity, and cell adhesion [76,77]. Dysregulation of the Hippo signaling pathway leads to cancer development. This dysregulation enables two central downstream effectors of Hippo signaling, Yes-associated protein (YAP) and its homolog protein TAZ, to translocate to the cell nuclei and serve as transcription factors that are considered major components involved in cancer (Scheme 2). As a result, research has been aimed at the development of pharmacological inhibitors to both YAP and TAZ, which serve as potent tumor drug targets. The search for physiological activators of YAP/TAZ led to the finding that GPCRs are actually powerful inducers of the YAP oncogenic pathway [78,79,80,81]. The tumor-suppressing Hippo pathway plays a major role in inhibiting YAP/TAZ nuclear localization and transcriptional activity, and the oncogenic YAP path is initiated upon abrogation of the Hippo path. Once the Hippo enzymatic cascade of events is inhibited, YAP/TAZ are dislodged from their cytoplasmic anchorage site and translocate to cell nuclei. In the nuclei, they serve as transcription co-activators and stimulate downstream target genes, consequently inducing oncogenicity through binding to TEAD family transcription factors.

The Mst1/2-Lats1/2 kinase cascade of the Hippo pathway inhibits YAP/TAZ through direct phosphorylation, leading to cytoplasmic retention via the binding of 14-3-3, which further promotes β-TrCP-mediated YAP/TAZ ubiquitination and degradation. GPCRs that are involved in cell proliferation are in fact capable of stimulating transcriptional activity of the co-activator YAP [76,80,82,83,84]. It has been shown that GPCRs inhibit the activity of LATS via Gα12/13, thus releasing YAP from LATS-dependent inhibition [80]. Studies from the Gutkind lab [85] have demonstrated that oncogenic mutations in Gαq lead to the activation of YAP by a mechano-sensing pathway as well as actin polymerization and not by intervention in the Hippo-suppressing pathway. In addition, recent studies from the Guan lab [86] have described YAP/TAZ as *bona fide* downstream effectors of the noncanonical Wnt signaling pathway. It was proposed that Wnt5a/b and Wnt3a induce YAP/TAZ activation independent of canonical Wnt/β-catenin signaling, and instead via the noncanonical Wnt-YAP/TAZ signaling axis, consisting of Wnt-FZD/ROR Gα12/13-Rho GTPases-Lats1/2, to promote stimulation of oncogenic YAP/TAZ- and TEAD-mediated gene transcription.

The regulatory mechanisms controlling YAP and TAZ activity appear to vary between tissues. Based on the fact that induced transcriptional activities of YAP/TAZ are centrally involved in cancer, attenuation of YAP and/or TAZ is a rather logical approach for the treatment and prevention of a wide array of malignancies. One approach could be reducing YAP dosage by *sh*RNA depletion. Screens for *sh*RNA-induced lethality in a large panel of human cancer cell lines showed that tumor cell lines activated for WNT signaling are specifically sensitive to the knock-down of YAP [87]. Thus, Rosenbluh et al. [87], underscore the concept that YAP inhibition does not necessarily correlate with levels of YAP activity, and may involve important TEAD-independent interactions mediated via YAP that may be critical for some types of cancer cells.

## 9. Protease-Activated Receptors, PARs, and Cancer

Proteinases and their inhibitors [88] account for over 2% of human genes. While proteases act by both mechanisms of nonreceptor- and receptor-mediated functions, the fact that they represent a significant percentage of the genome indicates their importance in regulating diverse tissue functions. Cell signaling may be controlled also via digestion of a wide spectrum of zymogens such as kininogens, chemokines, prohormones, and growth factor receptors, for example insulin receptors as well as cytokine precursors. It has also been known for over 40 years that the proteolytic enzymes thrombin and trypsin can induce cell proliferation through cell surface receptor activation, much like traditional growth factors such as epidermal growth factor and insulin [89,90,91,92], although the mechanistic details were not understood until sometime later. After an exhaustive search for a receptor that mediates functional responses of the main protease in the coagulation cascade, thrombin, yielded a “thrombin receptor”, which acts to induce platelet aggregation as well as cultured cell proliferation. This receptor was called protease-activated receptor, or PAR [93,94]. PARs form a subfamily within the larger GPCRs of rhodopsin-like class A GPCRs, and include four members: PAR_1_, PAR_2_, PAR_3_, and PAR_4_ [95]. PAR_1_, the first and prototype member of the family, mediates the response to thrombin signaling in most cell types, and was thus designated as a “thrombin receptor” [94]. While PAR_3_ and PAR_4_ provide a “back-up” system to PAR_1_ [96,97,98], PAR_2_ is not considered as a thrombin receptor” but is rather activated via a trypsin serine-protease and also by proteases that present upstream to thrombin [99]. PARs are activated via enzymatic digestion of the N-terminal extracellular portion, which gives rise to newly exposed ligands that act through intramolecular binding to extracellular loop number two for signal transmission [100]. Once activated, conformational changes transmitted via the transmembrane ™ domains to the cytoplasmic tails enable the binding association with α subunits of G proteins localized within the membrane, inside the cell compartment [101].

## 10. Novel Signaling of PARs Endowing Critical PH-Domain Binding Motifs

Although a growing number of roles for PAR_1&2_ in oncogenesis have been identified, the basic signaling machinery has not yet been elucidated. Signal-associating motifs in PAR_1&2_ C-tails have been shown to be essential for breast cancer development [102] through binding of signal proteins that possess a pleckstrin-homology (PH)-domain. These include Akt/PKB-PH domain as well as Etk/Bmx and Vav_3_, all of which can potently associate with both PAR_1_ and PAR_2_. The association takes place in a hierarchical manner, whereby priority is attributed to Etk/Bmx. A point mutation in H349APAR_2_, but not in R352A, potently inhibits PH-protein binding and is sufficient to markedly eliminate PAR_2_-induced breast tumor growth in vivo and placental extravillous trophoblast (EVT) invasion in vitro. Along this line, the PAR_1_ mutant *hPar1-7A*, which is incapable of associating with the PH domain, markedly inhibits mammary tumor development and EVT invasion, demonstrating the physiological significance and importance of these novel PAR_1_ and PAR_2_ PH domain binding motifs in both pathological and normal invasion processes. PH domains that are present in diverse signal transducing proteins are highly preserved motifs. They function as versatile modules in *protein-protein* interactions, inducing a multitude of physiological events [103,104]. These associating motifs are required for both tumor development and physiological placental EVT-uterus interactions. In spite of the fact that primary sequence identity between PH domains is limited, striking similarity is found in their tertiary structures.

Interestingly, the binding motifs in PARs [102] are either lipid-independent and mediated via protein-protein association, as demonstrated for PH-Etk/Bmx, or lipid-dependent, as is the case of Akt-PH association. One possible explanation is that membrane targeting is mediated via palmitoylation of a cysteine residue in the PAR_1&2_ C-tail. The previously identified, conserved eighth helix (H8) found in rhodopsin is located within the C-tail of PAR_1_ and PAR_2_, as in other receptors that belong to Class A. Specifically, the PAR_1_ PH domain binding site is found within the H8 loop. Likewise, palmitoylation of PAR_2_ is required for post-translational modification and is necessary for potent cell surface expression and desensitization of PAR_2_.

**Scheme 3:**
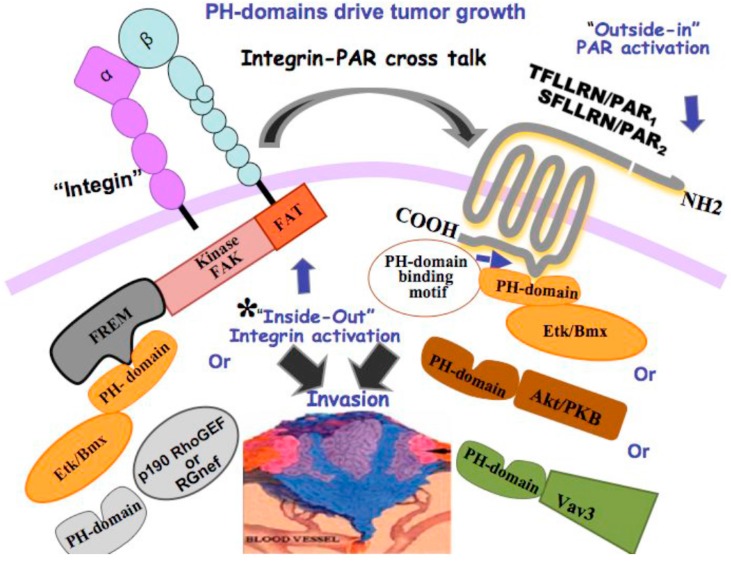
PH domains power tumor growth. The activation of PAR_1&2_ results in binding of PH signal proteins. These association motifs are essential for tumor development. The “inside-out” mode of integrin activation via either PH-Etk/Bmx-FERM/FAK or PH-Rgnef-FAK can induce interactions with PARs through all stages of cancer development.

In general, regardless of whether binding occurs via lipids or directly through *protein-protein* interactions, PH motifs that serve as pivotal binding modules demonstrate that interactions between separate motifs in a signal protein support transmission of a biochemical signal and also ensure a robust response to developmental cues, with sufficient specificity at precisely the right time to protect against premature and disastrous induction of a cell fate alteration. Cell surface receptors that play a central role in cancer biology, for example PARs, act to effectively relay cell signaling by recruiting PH domain signal proteins. PARs may also activate integrins, and vice versa [105]. Formation of the FAK-PH-Etk/Bmx complex, as well as FAK-PH-Rgnef (e.g., binding via PH domain), may initiate signaling in an “inside-out” manner that consequently will affect the extracellular portion of integrins and “activate” them. Once activated, integrins can associate with PARs ([104], see Scheme 3), which then will transmit signaling in an “outside-in” fashion and associate with PH-signal proteins.

## 11. Concluding Remarks and Future Directions

GPCRs are known to activate different signaling pathways initiated by ligand binding (see Table 1 summarizing driver GPCR signaling in cancer). The prospect of biased agonism conferring selectivity of signaling via the binding of several ligands to the same receptor offers advantages in clinical settings. It is desired to help design drugs with fewer unfavorable side effects, specifically in designing a biased ligand that will have a weak activity under one pathway condition but a much stronger activity in another. While hypothetically all GPCRs should demonstrate biased signaling, until now several were found to have this property. It is still not understood how a variety of stabilized conformations of a given receptor give rise to different signaling pathways. This challenging mechanism is in its early phase and needs to be further explored. It remains to be conclusively determined whether changes in the receptor conformation are indeed the ground basis for biased agonism signaling. Among other approaches, for example, is the identification of new binding motifs within GPCR C-tails that may allow for the design of selective potent drugs. Such therapeutic medicaments are expected to inhibit diverse signaling pathways initiated by a signaling partner that harbors a specific “signal-motif” for association with GPCRs and initiation of a signaling cascade. For example, the “PH domain binding motifs” within PARs are effectively capable of associating with several PH signal-possessing proteins (e.g., Etk/Bmx, Akt and Vav). There is a plan to identify such PH-binding motifs within the spectrum of GPCRs for future effective drug design.

## Figures and Tables

**Table 1:** Cancer GPCRs and signaling pathways. Summary of cancer driver GPCRs and their signaling. As such, examples of GPCRs which are implicated in human cancer are listed. Lysophosphatidic acid receptors 1-6 (LPA1-6), protease-activated receptors (PAR_1&2_), Yes-associated protein (YAP), *Frizzled* receptors (Fz), parathyroid receptor1 (PTHR1), endothelin receptors A and B (ET_A_R and ET_B_R), endothelin1-3 (ET1-3), prostaglandin receptors (PE2, PE4), prostaglandin (PGE2), bradykinin receptor type 1 and 2 (B1R, B2R), sphingosine-phosphate receptor 1 (S1PR1).

Receptor	Ligand	Pathways
Lysophosphatidic acid Receptors (LPA1-6)	Lysophosphatidic acid	Rho-dependent pathway [37,106]
		β-Catenin stabilization [107,108]
		Kruppel-like factor 5 [109]
Protease activated receptors (PAR_1&2_) LPA	Thrombin, Trypsin, respectively, or TFLLRN (G12/13, PAR1) or SLIGKV (G12/13, PAR2) [79] Lysophosphatidic acid (Gαq) [80]	Hippo/YAP pathways via activation of Gα12/13-coupled receptors or Gαq. Inhibition of Hippo pathway (via the inhibition of Lats1/2 kinases ) results in activation of YAP co-transcriptional activity [110]
Frizzled (Fz) PAR1 Parathyroid receptor1 (PTHR1)	Wnt 3A (canonical pathway)	Canonical Wnt signaling stabilization of β-catenin [66,114] and its transcription activity
	Thrombin or TFLLRN [111,112]	
	PTH [113]	
Chemokine receptor (CXCR4)	CXCL12, SDF-1	PI3K, Akt, Src PIP2, IP3, Ras, Raf, ERK1/2, PLC, JNK [115]
PAR1 and PAR2	Thrombin or TFLLRN (PAR1) Trypsin or SLIGKV (PAR2)	PH domain signal partners such as Etk/Bmx or Akt [102] Gα12/13, Rho [24]
Endothelin receptors (ETAR and ETBR)	endothelin-1-3 (ET-1, ET-2, ET-3)	C-Src/cross talk with EGFR
		β-arrestin -1or-2 PDZRhoGEF and Rho A, C
		β-catenin stabilization [7,21,116]
Prostaglandin receptors (PE2, PE4)	PGE2	Cyclooxygenase (COX-2)pathway P13K (coupling to Gs) [117,118,119]
Bradykinin Receptor Type 1 and 2 (B1R, B2R)	Kinins	Gαq and Cross talk with EGFR Ras, Raf, ERK
Sphingosine1-phosphate receptor1 (S1PR1)	S1P	Ras-ERK, PI3K-Akt-Rac, Rho, STAT3 (coupling to Gαi) [120,121]
